# Quality and Reliability Analysis of YouTube Videos on Magnetic Resonance Imaging Claustrophobia

**DOI:** 10.7759/cureus.37648

**Published:** 2023-04-16

**Authors:** Fuldem Mutlu, Erbil Arik

**Affiliations:** 1 Radiology, Sakarya University Medical Faculty, Sakarya, TUR; 2 Radiology, Igdir State Hospital, Igdir, TUR

**Keywords:** gqs, discern, claustrophobia, mri, magnetic resonance imaging

## Abstract

Aim

Magnetic resonance imaging (MRI)-induced anxiety is not infrequent with a reported incidence as high as 37% and the rate of failed MRI imaging due to claustrophobia ranges between 0.5% and 14.5%. The objective of this study was to evaluate the quality and reliability of YouTube^TM^ videos on MRI claustrophobia.

Methods

Sixty-five videos were included in the final analysis. Video information analyzed included video length (minutes), video content, qualification of the video uploaders, time of upload, time since upload, the number of total views and the mean daily views, and like counts. We divided the videos according to the uploaders into professional and non-professional groups and further grouped the videos as useful and misleading. Data obtained from the videos were evaluated with three tools including subjective evaluation, Quality Criteria for Consumer Health Information (DISCERN), and Global Quality Scale (GQS) tools.

Results

The mean video duration was found as 4.14±4.45 minutes. The mean view count was 104.59±408,788.68 and the mean like count was found as 272.55±1096.25. Seventeen (26.15%) videos were uploaded by professionals and 48 (73.85%) by non-professionals. Twenty-eight (43.08%) of the videos were useful and 37 (56.92%) were useless. The mean DISCERN and GQS scores were statistically significantly higher in the professional videos compared to the non-professional videos and in useful videos compared to non-useful videos (for all, p<0.001).

Conclusion

A majority of the YouTube^TM^ videos concerning MRI claustrophobia were uploaded by non-professionals. Physicians and other healthcare personnel should be encouraged to provide useful and accurate videos and to direct patients appropriately.

## Introduction

Magnetic resonance imaging (MRI) was introduced in the field of medicine in the 1980s, and since then has become a popular, non-invasive imaging modality [1]. MRI provides 3D detailed anatomical images of the body without using ionizing radiation [2]. Today, MRI is one of the most commonly used diagnostic tools in the management of many diseases and to evaluate response to treatment [3].

Claustrophobia is defined as a marked, excessive, or unreasonable fear of being trapped in an enclosed space [4]. For a claustrophobic person, being in a confined space will almost invariably provoke fear and discomfort in the case of a mild phobia, or anxiety and panic attacks in the case of a more severe phobia [5]. MRI usually involves the patient being confined within a narrow tunnel-like structure and remaining motionless. MRI-induced anxiety is not infrequent with a reported incidence as high as 37% and the rate of failed MRI during claustrophobia ranges between 0.5% and 14.5% [6]. Although open MRI devices have been developed recently, fear of MRI remains a common phenomenon among the public. However, open MRI devices, which are useful for claustrophobic patients, cannot replace normal MRI in all cases.

As in many diseases and medical conditions, patients and/or their relatives tend to search for information from various sources before seeking professional healthcare aid. The internet is the leading source of information for this purpose with millions of health-related websites and a great number of sharing platforms, including social media. With the increasingly widespread use of sharing platforms on the Internet, today it is common for a physician to encounter patients who have already done their research on the Internet to find treatment options for their disease [7]. Currently, about 80% of the global population is using the Internet as a source of information to seek a remedy for their health problems, to share experiences as patients and/or their relatives and even to buy treatment online [8]. YouTube^TM^ (www.youtube.com) is the most frequently visited video-sharing platform. In 2021, there were approximately 1.86 billion global YouTube^TM^ users and this figure is estimated to increase to 210 million in 2022. More than 500 hours of videos are uploaded to YouTube^TM^ every minute. Videos can be uploaded to YouTube^TM^ at any time, by any person, and free of charge, making YouTube^TM^ a source of health-related information [9]. However, this has raised concerns about the reliability and quality of the videos on YouTube^TM^. These concerns are much more intense relative to misleading YouTube^TM^ videos on the ongoing coronavirus disease 2019 (COVID-19) pandemic. In addition, YouTube^TM^ has no filtering options or a clear policy for health-related misleading videos, prompting many studies of the quality and reliability of YouTube^TM^ videos in several diseases and medical conditions [10-15]. To the best of our knowledge, the literature contains no study that evaluates YouTube^TM^ video content specific to fear of MRI. The objective of this study was to evaluate the quality and reliability of YouTube^TM^ videos on MRI claustrophobia.

## Materials and methods

Study design and search strategy

**Figure 1:**
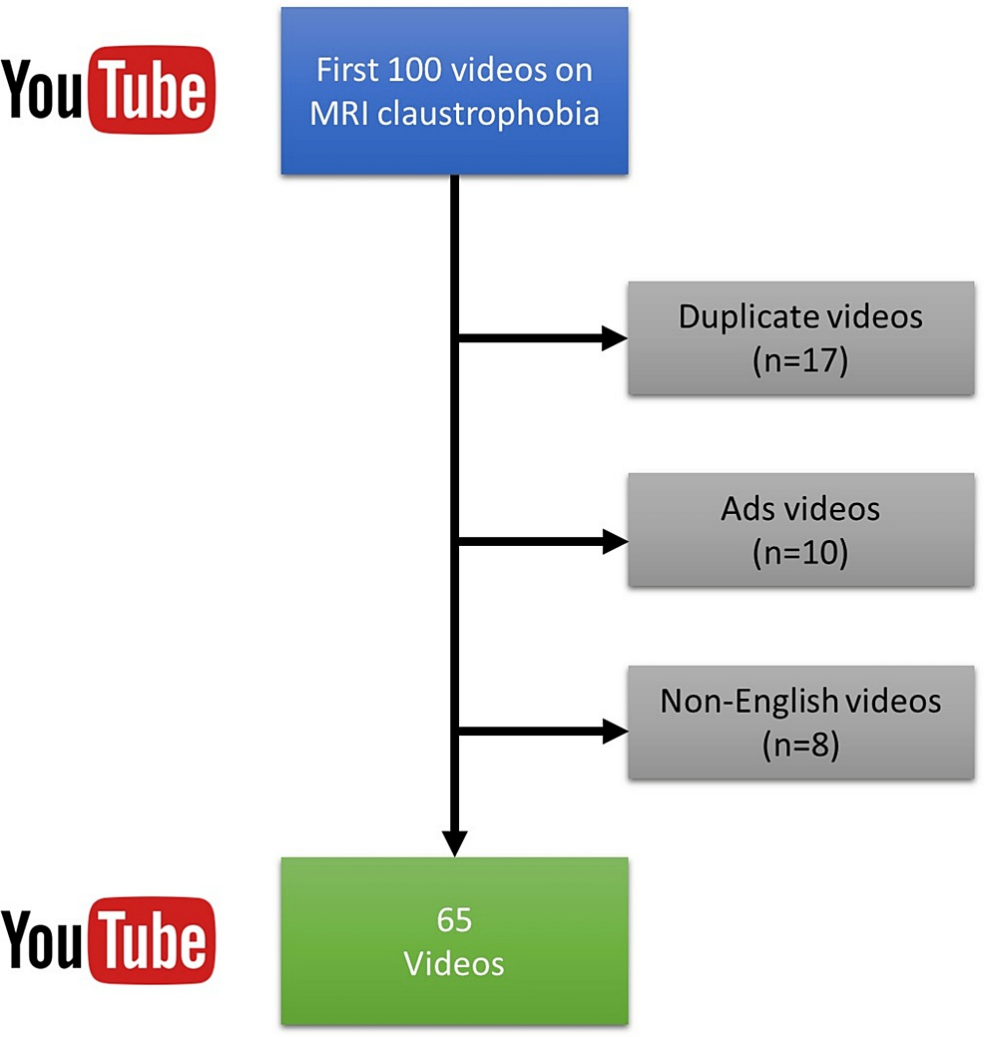
Flowchart of the videos included in the study.

This study was conducted by two independent experienced radiologists (14 years and three years) on the quality and reliability of YouTube^TM^ videos on fear of MRI, namely MRI claustrophobia, on January 24, 2023. In the case of disagreement, a third colleague (15 years) was invited, and the decision was made by the three. The search terms were determined by the two radiologists as “Fear of MRI,” “MRI phobia,” and “MRI claustrophobia.” The terms were entered into the search box of YouTube^TM^ and from the filtering feature, the “relevance” option was chosen. The first 100 videos returned by the search were subjected to the analysis because it has been reported that most YouTube^TM^ searchers view the first of the yielded results. Most previous studies have used the first 50 or 100 videos for YouTube^TM^ analysis [10-16]. Although some studies have evaluated all videos, the most commonly used methodology is to include a fixed sample [17]. Among the 100 videos, duplicate videos, ads, non-English videos, and those that were longer than 30 minutes were excluded from the study. The remaining 65 videos were included in the final analysis. The flowchart of the YouTube^TM^ videos is shown in Figure 1.

Data collection and evaluation

In order to extract information from the videos and make the assessment, a Microsoft Excel sheet was prepared by the researcher. Links to the 65 videos were entered into this file. Video information analyzed in this study included video length (minutes), video content, qualification of the video uploaders, time of upload, time since upload, the number of total views and the mean daily views, and similar counts. In order to make a more accurate comparison, the average daily view counts of the videos were calculated by dividing the number of total views by the time (in days) since the video was uploaded. In previous studies, Video Power Index (VPI) based on like and dislike counts has been used to evaluate the popularity of the videos. However, YouTube^TM^ has removed the dislike count and so we could not include VPI in our analysis.

We divided the videos into two principal groups according to whether the uploaders were professional or non-professional and further grouped the videos as useful and useless and/or misleading. Useful videos are those with scientifically accurate content helping patients to make an informed decision about treatment. Useless or misleading videos are those with scientifically inaccurate content that mislead patients regarding decisions for treatment. Professional videos consisted of the videos uploaded directly by doctors, MRI technicians, and hospital channels. Most of the hospital videos were narrated by doctors. Non-professional videos included those uploaded by patients, health channels, and others. Videos uploaded by health channels were included in this group because they mostly include informative content rather than focusing on the fear of MRI.

Data obtained from the videos were evaluated with three tools. First was a subjective evaluation made by mutual consensus of the two authors and the reviewed videos were classified as useful or misleading. YouTube^TM^ videos on the fear of MRI were further evaluated using the Quality Criteria for Consumer Health Information (DISCERN) and Global Quality Scale (GQS) tools. The two authors independently rated each video in separate rooms, but at the same time interval in order to prevent the influence of one author by the other author during scoring.

DISCERN scoring

The DISCERN is a scoring system used for the evaluation of the reliability of consumer healthcare information on treatment options. In this study, the modified DISCERN tool that was developed by Singh et al. was used [18]. The DISCERN scoring involves five items that are evaluated with a 5-point Likert scale that examines the aims, reliability of information sources, bias, areas of uncertainty, and additional sources. According to the modified DISCERN scoring, the reliability of video content is accepted as good for a DISCERN score > 3 points, moderate for a DISCERN score = 3 points, and poor for a DISCERN score < 3 points [11].

GQS scoring

The GQS, which was developed first by Bernard et al. [19], is a scoring system used to assess the quality of video content according to the usefulness of the information. GQS includes five items examining the quality and ease of use of the viewed videos based on a 5-point Likert scale. A GQS score of 1 point is considered very poor, 2 points as poor, 3 points as moderate, 4 points as good, and 5 points as excellent.

Ethics considerations

Ethics approval was waived because no humans or animals were included in the study. In addition, permission from YouTube^TM^ was also waived, since all videos used in this study were publicly available. However, the study was conducted in line with the ethical principles of the Declaration of Helsinki.

Statistical analysis

The data obtained in this study were analyzed using SPSS version 25.0 (SPSS, Statistical Package for Social Sciences, IBM Inc., Armonk, NY, USA) statistical software. The normality of the data was analyzed with the Kolmogorov-Smirnov test. Since the variables were non-normally distributed, a comparison of the continuous variables between the two groups was made using the Mann-Whitney U test. Categorical variables were compared using the Chi-square test. The continuous variables were expressed as the mean ± standard deviation and the categorical variables as frequency (number, percentage). Cronbach alpha coefficients were used to calculate the agreement between the two raters. p<0.05 values were considered statistically significant.

## Results

After the excluded videos, the remaining 65 YouTube^TM^ videos on MRI claustrophobia were analyzed and evaluated in terms of quality and reliability by two radiologists, independently of each other. Video content was found as general information on MRI in 20 (30.77%), sharing experience in 17 (26.25%), and overcoming fear of MRI in 28 (43.08%) videos. The mean video duration was found as 4.14±4.45 minutes. The mean view count was 104.59±408,788.68. The oldest video was uploaded on February 10, 2010 and the latest one on December 6, 2021. The mean like count was found as 272.55±1,096.25. The most liked video was uploaded by a health channel to relate general information on MRI and received 8,200 likes, while eight (4.85%) received no like. The main characteristics of the reviewed videos are given in Table 1.

**Table 1:** Main features of the videos Min: Minute; MRI: Magnetic resonance imaging; SD: standard deviation

Parameter	mean	± SD
Video length (min)	4.27	4.54
View count	102.98	405789. 69
Daily view count	195.61	1250.33
Like count	268	1088.17
	n	%
Video content		
General information	20	30.77
Experience	17	26.25
Overcome fear	28	43.08
Uploaders		
Doctors	7	10.77
MRI technicians	5	7.69
Hospital channels	5	7.69
Health channels	18	27.68
Patients	18	27.68
Others	12	18.46

**Figure 2:**
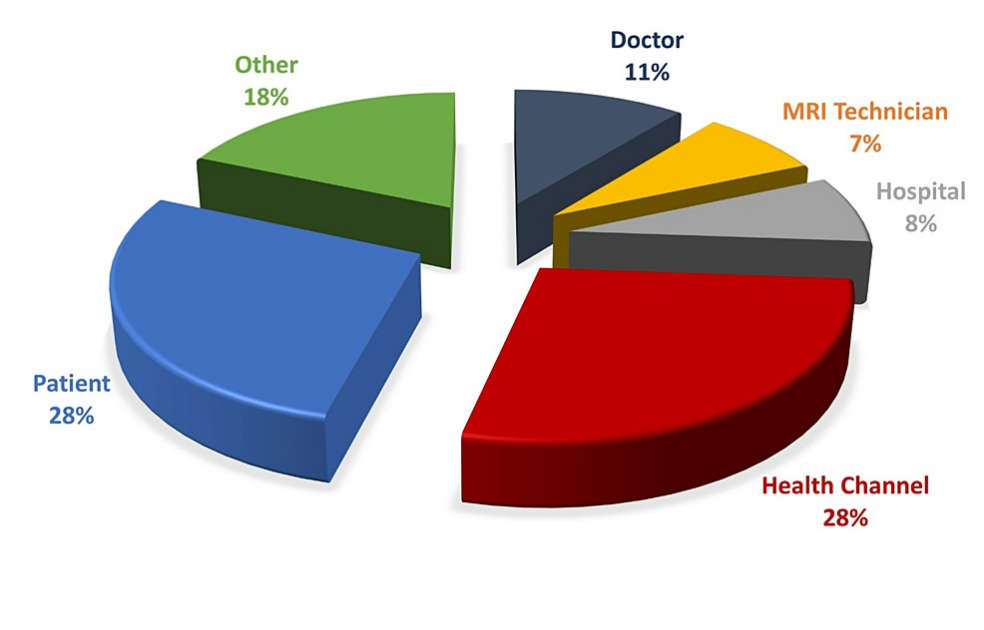
Distribution of video uploaders

Qualifications of the video uploaders are given in Figure 2.

**Figure 3:**
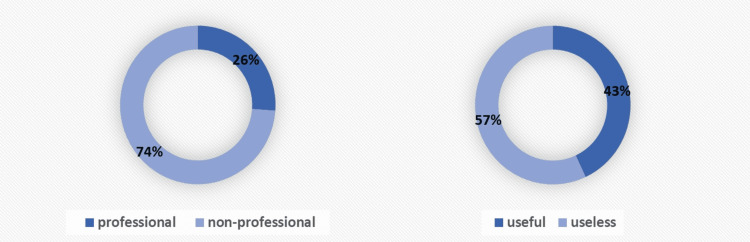
Grouping of the videos

The video providers were assigned to two groups - professionals and non-professionals. The professionals group included doctors, MRI technicians, and hospitals. Hospitals were included in this group because the vast majority of these videos were narrated by doctors. Health channels were included in the non-professional group because the videos uploaded by these channels mostly involved general information on MRI and did not direct patients for coping with anxiety occurring due to MRI. Accordingly, 17 (26.15%) videos were uploaded by professionals and 48 (73.85%) by non-professionals. The videos were divided into two groups as useful and useless through a subjective assessment made by mutual consensus of the two authors. Accordingly, 28 (43.08%) of the videos were useful and 37 (56.92%) were useless (Figure 3). The study parameters, DISCERN, and GQS scores were compared between these paired groups.

The mean video length was found as 2.69±2.18 minutes in the professional and 4.63±4.91 minutes in the non-professional videos, with no statistically significant difference found between the groups (p=0.215). The mean view count was 99,655.81±193,330.10 in the professional and 106,236.50±460,495.76 in the non-professional videos (p=0.317). The mean like count was 273.69±762.03 in the professional and 272.17±1,193.96 in the non-professional videos (p=0.920).

The mean video length was found as 3.27±3.69 minutes in the useful and 5.02±5.01 minutes in the useless videos, with no statistically significant difference found between the groups (p=0.233). The mean view count was 184,630.79±603,435.15 in the useful videos and 41,195.38±102,611.27 minutes in the useless videos (p=0.827). The mean like count was 405.11±1,558.40 in the useful videos and 164.92±508.02 minutes in the useless videos (p=0.279).

The mean DISCERN and GQS scores were statistically significantly higher in the professional videos compared to the non-professional videos and in useful videos compared to non-useful videos (for all, p<0.001). The mean DISCERN and GQS scores according to the groups are given in Table 2.

**Table 2:** DISCERN and GQS scores according to the groups DISCERN: Quality Criteria for Consumer Health Information; GQS: Global Quality Scale

	n (%)	DISCERN	GQS	p value
		mean ± SD	mean ± SD	
Uploaders				
Professionals	17 (26.15)	4.06±1.12	4.13±1.01	p<0.001
Non-Professional	48 (73.85)	2.90±0.88	3.08±0.85	
Usefulness				
Useful	28 (43.08)	4.04±0.88	4.09±0.85	p<0.001
Useless	37 (56.92)	2.73±0.66	2.77±0.64	

**Figure 4:**
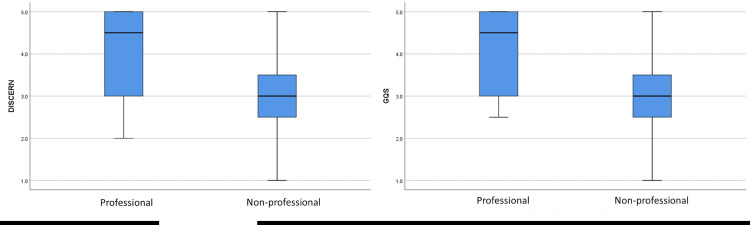
The mean DISCERN and GQS scores between the professional and non-professional videos.

Figure 4 shows the mean ± standard deviations of DISCERN and GQS scores between the professional and non-professional groups.

According to DISCERN scale, the reliability of the reviewed videos was considered poor in 21 (32.31%) videos, moderate in 18 (27.69%), and good in 27 (41.54%) videos. Agreement between the two radiologists in the evaluation of the videos was assessed using the Cronbach alpha coefficient. There was an excellent agreement between the two researchers (Table 3).

**Table 3:** Agreement between the observers DISCERN: Quality Criteria for Consumer Health Information; GQS: Global Quality Scale; SD: standard deviation

	Mean ± SD	P-value	r	Cronbach α
DISCERN 1	3.18±1.06	p<0.01	0.854	0.867
DISCERN 2	3.37±0.99			
GQS 1	3.34±1.06	p<0.01	0.906	0.886
GQS 2	3.32±1.00			

## Discussion

In this study, we evaluated the quality and reliability of YouTube^TM^ video contents on MRI claustrophobia, which has not been largely eliminated by open MRI as open MRI cannot replace conventional MRI in many cases, but remains an important problem, because (1) many non-open MRI devices remain in use and (2) some people who have fear of MRI and experience panic attack and/or anxiety during MRI procedure. In our study, the mean length of videos was found as 4.14 minutes. Looking at recent YouTube^TM^ analysis studies in the literature, the mean video length was reported as 7.56 minutes by Kuru and Erken [16] for YouTube^TM^ videos on rotator cuff tears, 8.10 minutes by Ku et al. [20] for videos on male infertility, 5.25 minutes by Aydin and Yilmaz [21] for videos on echocardiography.

In our study, the most common video content was directed at overcoming fear of MRI or MRI claustrophobia followed by general information and sharing experience. It is obvious that video content widely differs among studies depending on the objectives and the topic of study. We think that experience sharing is a common content item among YouTube^TM^ analysis studies [22,23]. In the present study, no significant difference was found between the videos uploaded by professionals and those uploaded by non-professionals in terms of the like counts. Similarly, there was no statistically significant difference between the useful and useless studies in terms of the like counts.

We divided the videos into two groups as the videos were uploaded by professionals (doctors, MRI technicians, and hospitals) and those uploaded by non-professionals (health channels, patients, others). In addition, we divided the videos into further two groups as useful and useless videos based on consensus subjective evaluation by the two authors. Similarly, in a study by Onder and Zengin [8] evaluating YouTube^TM^ videos on psoriatic arthritis, the videos were evaluated as useful and misleading. Jamleh et al. [24] analyzed YouTube^TM^ videos on periradicular surgery and examined the videos with usefulness scores as poor, moderate, and good. Kocyigit and Akyol [25] investigated YouTube^TM^ videos on COVID‑19 vaccination in rheumatic diseases and divided the videos into three groups based on the quality scores as low, intermediate, and high-quality videos.

In the present study, we evaluated videos through DISCERN and GQS scales. Both scores were statistically significantly higher in the professional group compared to the non-professional group and in the useful group compared to the useless group (for both, p<0.001). There are several reasons for these findings. First, videos uploaded by patients mostly contained experience sharing, explanations of fearful times during MRI and even increasing fear of the audience. Videos uploaded by health channels did not give information on how to overcome MRI claustrophobia; instead, they introduced the modern MRI devices that they use, and although these videos were not ads or misleading, they were useless for the topic of our study [26]. In a YouTube^TM^ analysis study on gout, Onder and Zengin [8] reported the mean DISCERN as 3 and GQS as 4 for overall videos. In the same study, the mean DISCERN score was 3.0 for useful and 2.0 for misleading videos while the mean GQS score was reported as 4.0 for useful and 2.0 for misleading videos. In another study, Chang and Park reported the mean DISCERN score as 1.6 for low-quality, 3.0 for intermediate-quality, and 3.39 for high-quality YouTube^TM^ videos on Compensated Maneuvers for Dysphagia. In a study analyzing YouTube^TM^ videos on the Surgical Treatment of Uterine Leiomyomas, Ergul [27] reported the mean DISCERN score as 1.6±0.9 for patient experience. In the present study, we found the mean DISCERN score for videos including patient experience as 2.87±1.72. Our higher score compared to the previous studies may be attributed to the difference between the topics of the studies.

Finally, according to DISCERN scale, the reliability of the reviewed videos was considered poor in 21 (32.31%) videos, moderate in 18 (27.69%), and good in 27 (41.54%) videos. In a study by Karakoyun and Yildirim [28] evaluating YouTube^TM^ videos about Behçet’s disease, 46% of the videos were found to have low-to-moderate reliability and 56% good quality. In the present study, as high as 73.85% of videos pertaining to the fear of MRI were uploaded by non-professionals with most of them being patients. This suggests a significant difference in quality and reliability for videos uploaded by professionals and non-professionals. Studies on YouTube^TM^ videos in the literature are largely consistent with the present study.

Study limitations

This study has some limitations. The study involved a snap-shot assessment of the reviewed videos at a certain period of time. Whereas these videos can be uploaded or removed at any time. In order to minimize subjective bias in video scoring process, further studies may include different observers of various backgrounds such as different age groups and healthcare consumers, i.e., patients. However, we think that our results will be a preliminary guide for future studies in terms of patient education on imaging methods and this is the first study to evaluate YouTube^TM^ videos on MRI claustrophobia.

## Conclusions

According to the findings of our study, a majority of the YouTube^TM^ videos concerning fear of MRI or MRI claustrophobia were uploaded by non-professionals with poor quality and reliability. Physicians and other healthcare personnel should be encouraged to provide useful and accurate videos and to direct patients appropriately. Healthcare-related YouTube^TM^ videos should be supervised by YouTube^TM^ and regulatory bodies and be classified as professional or non-professional videos before they are made accessible to the public. 
